# Aspirin, metformin, and statin use on the risk of gastric cancer: A nationwide population‐based cohort study in Korea with systematic review and meta‐analysis

**DOI:** 10.1002/cam4.4514

**Published:** 2021-12-30

**Authors:** Seung In Seo, Chan Hyuk Park, Tae Jun Kim, Chang Seok Bang, Jae Young Kim, Kyung Joo Lee, Jinseob Kim, Hyon Hee Kim, Seng Chan You, Woon Geon Shin

**Affiliations:** ^1^ Department of Internal Medicine Kangdong Sacred Heart Hospital Hallym University College of Medicine Seoul Korea; ^2^ Institute for Liver and Digestive Diseases Hallym University Chuncheon Korea; ^3^ Department of Internal Medicine Hanyang University Guri Hospital Hanyang University College of Medicine Guri Korea; ^4^ Department of Internal Medicine Samsung Medical Center Sungkyunkwan University School of Medicine Seoul Korea; ^5^ Department of Internal Medicine Chuncheon Sacred Heart Hospital Hallym University College of Medicine Chuncheon Korea; ^6^ University Industry Foundation Hallym University Chuncheon Korea; ^7^ Department of Epidemiology School of Public Health Seoul National University Seoul Korea; ^8^ Department of Statistics and Information Science Dongduk Women's University Seoul Korea; ^9^ Department of Preventive Medicine Yonsei University College of Medicine Seoul Korea

**Keywords:** aspirin, chemoprevention, gastric cancer, metformin, statin

## Abstract

**Background/Aims:**

Although several chemopreventive drugs against gastric cancer have been proposed, their effects have not been fully evaluated. We examined the impacts of aspirin, metformin, and statin use on gastric cancer development in a population‐based cohort in Korea.

**Methods:**

We analyzed the association between potential chemopreventive drugs—aspirin, metformin, and statin—and gastric cancer through the Observational Medical Outcomes Partnership Common Data Model‐based Korean nationwide cohort. Use of aspirin, metformin, and statin was defined by ≥365 days of prescriptions for each drug in the general population. To summarize the current evidence, we further performed a systematic review and meta‐analysis of the impact of aspirin, metformin, and statin on gastric cancer development.

**Results:**

After propensity score matching, 31,839, 6764, and 10,251 subjects were observed for medians of 4.7, 4.2, and 4.2 years for aspirin, metformin, and statin analysis, respectively. Use of aspirin or statin was associated with lower risks of gastric cancer compared to their non‐use, respectively (hazard ratio [HR] [95% confidence interval [CI]]: aspirin, 0.72 [0.60–0.85], *p* < 0.01; statin, 0.67 [0.49–0.92], *p* = 0.01). However, no association was observed between metformin use and gastric cancer development (HR [95% CI]: 0.85 [0.59–1.23], *p* = 0.40). A subgroup of subjects with diabetes mellitus showed a lower risk of gastric cancer development with statin use. The meta‐analysis showed the highest effect size of gastric cancer development for statin, followed by aspirin and metformin.

**Conclusions:**

Statin and aspirin use were associated with significantly reduced risks of gastric cancer development, while the use of metformin was not associated with the gastric cancer risk. The protective effect of statin against gastric cancer was also significant in patients with diabetes mellitus.

## Graphical abstract

Statin and aspirin use were associated with significant reductions in the risk of gastric cancer development, while the use of metformin was not associated with the gastric cancer risk. The protective effect of statin against gastric cancer was also significant in patients with diabetes mellitus.

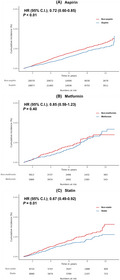

## 1 INTRODUCTION

Gastric cancer is the sixth most common cancer and the third‐leading cause of cancer death worldwide, with the highest incidence rates especially in Eastern Asia.^1^ The primary prevention of gastric cancer includes reducing intake of foods preserved by salting; increasing consumption of fresh fruits and vegetables; not smoking; and treatment of *Helicobacter pylori* (*H*. *pylori*) infection.^2^ Although *H*. *pylori* eradication therapy is well known as the most definitive method for chemoprevention of gastric cancer, it cannot fully reduce the risk of gastric cancer.^3^

The chemopreventive effects of cardiovascular or anti‐diabetic medications such as aspirin, metformin, and statin against gastric cancer have been proposed.^4^, ^5^, ^6^, ^7^, ^8^, ^9^ Aspirin acts as a chemopreventive agent by cyclooxygenase‐2 (COX‐2) inhibition and non‐cyclooxygenase pathways such as phosphatidylinositol 3‐kinase, nuclear factor‐κB, Wnt‐ß‐catenin, extracellular signal‐regulated kinase, and activated protein 1.^4^ Possible anti‐carcinogenic mechanisms of metformin include direct activation of the 5′ adenosine monophosphate‐activated protein kinase (AMPK) pathway and indirect effects from lowering the blood glucose and insulin levels.^7^ Statins have shown potential chemopreventive effects on various solid tumors, which are believed to be mediated by arresting cell cycle progression inducing apoptosis, inhibiting angiogenesis, and immunomodulation.^10^ However, those studies were based on heterogeneous populations and study designs. Besides, the effect of drugs on gastric cancer might differ by region, ethnicity, and regional prevalence of *H*. *pylori* infection. Nonetheless, few studies have assessed the drug effects on gastric cancer in countries with high incidence of gastric cancer, adjusting various confounding factors. Moreover, the chemopreventive effects of aspirin, metformin, and statin were evaluated in different studies, separately, not in the same database. Generally, these medications often could be co‐prescribed in clinical practice, therefore, it is needed to evaluate the chemopreventive effects of each drug in the same database with adjusting many factors that might affect the gastric cancer.

Therefore, this study aimed to identify whether aspirin, metformin, and statin are associated with gastric cancer prevention using a nationwide population‐based cohort in Korea, where gastric cancer is highly prevalent. We additionally conducted the systematic review and meta‐analysis to validate our study findings.

## 2 METHODS

### 2.1 Study design

This nationwide population‐based cohort study evaluated the association between potential chemopreventive drugs including aspirin, metformin, and statin on the risk of gastric cancer through the Observational Medical Outcomes Partnership Common Data Model (OMOP‐CDM)‐based Korean database retrospectively. The analyses were performed using Observational Health Data Science and Informatics (OHDSI), an international, open‐science collaborative of more than 220 health care organizations supporting large‐scale observational research with OMOP‐CDM.^11^ To validate our findings, we conducted the systematic review and meta‐analysis of previously published studies. The protocol of the current study was approved by the Institutional Review Board of Kangdong Sacred Heart Hospital (IRB no. 2019‐05‐014).

### 2.2 Database

We obtained data from the National Health Insurance Service–National Sample Cohort (NHIS–NSC), a Korean nationwide cohort comprising about one million subjects.^12^ Korea has a single‐payer health insurance system managed by the NHIS. In 2002, the NHIS established cohort data representative of the Korean population for research purposes.^12^ These data included a demographic profile, health insurance claims data, death registry, disability registry, and national health check‐up data. The NHIS–NSC data during 2002 and 2013 were converted into the OMOP‐CDM model.^13^ The converting process was described previously,^13^ and OMOP‐CDM‐based studies have been validated in multiple studies.^14^, ^15^, ^16^, ^17^

### 2.3 Study population and cohort definitions

To identify the impact of aspirin, metformin, and statin on gastric cancer development, we selected target (drug user) and comparator (non‐drug user) cohorts from a general population; namely, all subjects included in the NHIS–NSC. As part of subgroup analysis, we also analyzed data using additional target and comparator cohorts in a diabetes mellitus (DM) population because these drugs are common medications used by patients with DM.

The target cohort was defined as subjects who were prescribed the target drugs (aspirin, metformin, or statin) for more than 365 days. The included concept identifications associated with the target drugs are listed in Table S1. The index date was defined as the date of the first prescription of the target drug (exposure). The gaps of less than 30 days between drug prescription were permitted, and it were regarded as continuous drug exposures. The comparator cohort was defined as subjects who were prescribed any other drug excluding each target drug (aspirin, metformin, or statin) for more than 365 days. The target and comparator cohorts were censored if they were diagnosed with gastric cancer or the observation period ended in the database. Subjects who met at least one of the following criteria were excluded from both target and comparator cohorts: (1) history of any previous malignant neoplasm beyond the exposure ascertainment period of 3 years before cohort entry; (2) an observation time of less than 1 year prior to cohort entry; and (3) age <18 years at cohort entry. The construction of cohorts is presented in Figure S1.

Within the target and comparator cohorts, subjects with DM were selected based on the International Classification of Diseases (ICD)‐10 codes. The target cohort in the DM population was defined as subjects who received target drugs for 30 days or more within 1 year of DM diagnosis. The comparator cohort was defined as subjects who had been exposed to oral hypoglycemic agents or insulin within 1 year of DM diagnosis with no exposure to target drugs. The censoring rules were applied equally with general population.

The outcome cohort was defined as subjects who were newly diagnosed with gastric cancer after 1 year from the index date. Gastric cancer was identified using the ICD‐10 diagnosis codes C16.0–C16.9, and D002 (carcinoma in situ of stomach).

### 2.4 Outcomes

The primary outcome was the impact of aspirin, metformin, and statin on gastric cancer prevention in a general population. The secondary outcome was the impact of those drugs on gastric cancer prevention in DM patients.

### 2.5 Statistical analysis

OHDSI research provides large‐scale propensity score models with regularized logistic regression. The following covariates were used for propensity score matching between the target and comparator cohorts: age, sex, index year, all previous comorbidities, all drugs in the 365 days prior to the index date, and Charlson comorbidity index. Of those who had health check‐up, smoking history, alcohol consumption, body weight, and family history of cancer were included in covariates. To adjust for previous *H*. *pylori* eradication therapy between the target and comparator cohorts, prescribed drugs for *H*. *pylori* eradication before the index date, including proton pump inhibitors, amoxicillin, clarithromycin, bismuth, tetracycline, and metronidazole, were also included as covariates. Propensity score matching was performed in a 1:1 ratio and a caliper of 0.25 on the logit scale. Propensity score was estimated using logistic regression models with the L1 penalty hyper‐parameter selected through 10‐fold cross‐validation using high‐performance computing.^18^

We developed Cox proportional hazard models to calculate the hazard ratios (HRs) with 95% confidence intervals (CIs) for the risk of gastric cancer development between the target and comparator cohorts, using the CohortMethod package in R. The Kaplan–Meier method was used to estimate the cumulative incidence rates, and the cumulative incidence between two groups was compared using the log‐rank test. To evaluate the robustness of the main analysis results, multiple sensitivity analysis was conducted with various case–control ratios and lag periods. Empirical calibration of the *p*‐values was conducted by fitting an empirical null distribution to point estimates of negative control outcomes, which were expected to not be related with the target or comparator cohort and assumed that true relative risk between the target and comparator cohorts was 1.^19^ Ninety selected negative control outcomes are listed in Table S2.

Two‐sided *p*‐values <0.05 were considered statistically significant in all comparisons. All analyses were performed using ATLAS ver. 2.7 and R statistical software (version 3.6.1 for Windows; R Foundation for Statistical Computing).

### 2.6 Systematic review and meta‐analysis

For the systematic review and meta‐analysis, we searched the MEDLINE, EMBASE, and Cochrane Library databases for all relevant studies published between 1980 and 2020 that examined the risk of gastric cancer development according to the administration of aspirin, metformin, or statin. The detailed search strategies and latest search date are shown in Appendix S1.

The inclusion criteria were (a) population: adults in community‐ or hospital‐based cohorts; (b) intervention: administration of aspirin, metformin, or statin; (c) comparator: no administration of aspirin, metformin, or statin; (d) outcome: gastric cancer development; and (e) study design: cohort or case–control studies. Non‐original studies, non‐human studies, abstract‐only publications, and studies published in languages other than English were excluded.

We first reviewed the titles and abstracts of the studies identified by our keyword search. Duplicates from multiple databases were removed, and irrelevant studies were then excluded according to the inclusion and exclusion criteria. Finally, we screened the full texts of the remaining studies. Two investigators (S.I.S. and C.H.P.) independently evaluated the studies for eligibility. Any disagreements were resolved through discussion and consensus. If agreement could not be reached, a third investigator (W.G.S.) determined the final eligibility.

Data were extracted using a data extraction form that had been developed in advance. Two investigators (S.I.S. and C.H.P.) independently extracted information on the first author; year of publication; study design; country; study period; publication language; definitions of aspirin, metformin, or statin use; and the risk of gastric cancer development according to the groups. As the primary outcome of the meta‐analysis, we analyzed the pooled risk of gastric cancer development according to the type of medication (aspirin, metformin, and statin) based on all relevant studies. Then, we further demonstrated the risk of gastric cancer development in studies of diabetic patients only, if possible. Besides, subgroup analyses according to study regions (Eastern vs. Western studies) were performed.

For the meta‐analysis, pooled odds ratios (ORs) or HRs with 95% CIs were calculated. A random‐effects model was utilized in the meta‐analyses. We assessed heterogeneity using two methods: Cochran's *Q* test, wherein *p*‐values <0.1 were considered statistically significant for heterogeneity, and *I* ^2^ statistics, wherein values >50% were suggestive of significant heterogeneity.^20^ All *p*‐values were two‐tailed, and *p*‐values <0.05 were considered statistically significant in all tests in the meta‐analyses except for heterogeneity. Analysis and reporting were performed in accordance with the Preferred Reporting Items for Systematic Reviews and Meta‐Analyses (PRISMA) guidelines.^21^ All meta‐analyses were conducted using the statistical software Review Manager 5.4 (version 5.4.0; Cochrane Collaboration, Copenhagen, Denmark) and R (version 4.0.2; R Foundation for Statistical Computing, Vienna, Austria).

## 3 RESULTS

A total of 1,025,340 subjects in the NHIS–NSC were eligible in our study. The attrition flow charts of each drug are summarized in Figure S2. After large‐scaled propensity score matching, a total of 31,839, 6764, and 10,251 subjects were included in the final analysis for aspirin, metformin, and statin, respectively. The covariates balance before and after propensity score matching are presented in Figure S3. Overall, the differences in covariates before propensity score matching were well resolved after matching. To assess for systematic errors in our study populations, the relative risks of the target drugs (aspirin, metformin, and statin) for negative control outcomes were plotted in funnel plots (Figure S4). The plots showed no asymmetry and most negative control outcomes showed no significant difference between two cohorts. The baseline subject characteristics in the target and comparator cohorts are described in Table 1. The most common medical history was hypertensive disorder for aspirin user, DM for metformin user, and hyperlipidemia for statin user. The most common medication was antibacterial use, followed by drugs for acid‐related disorders in all analyses. We adjusted for medications that may alter the risk of gastric cancer including aspirin, metformin, statin, and nonsteroidal anti‐inflammatory drugs (NSAIDs), and the covariates were well matched (Table 1). Of the anti‐inflammatory drugs and antirheumatic products, the detailed proportion of each drug of NSAIDs is presented in Table S3.

**TABLE 1:** Baseline characteristics in the analysis of aspirin, metformin and statin

Characteristic,%	After propensity score adjustment								
	Aspirin (*n* = 31,839)	Non‐aspirin (*n* = 31,839)	SMD	Metformin (*n* = 6764)	Non‐metformin (*n*=6,764)	SMD	Statin (*n* = 10,251)	Non‐statin (*n* = 10,251)	SMD
Index year									
2003	12.5	12.9	−0.01	11.8	14.4	−0.08	9	9.3	−0.01
2004	10.8	10.8	0.00	9.8	9.9	0	7.6	7.7	0
2005	11.1	11.2	0.00	9.6	9.7	0	8.8	9.1	−0.01
2006	10.6	10.7	−0.01	8.2	7.8	0.02	8.6	8.9	−0.01
2007	10.0	9.8	0.01	8.7	8.4	0.01	9.1	8.7	0.01
2008	9.7	9.8	0.00	8.6	7.5	0.04	9.4	9.6	−0.01
2009	8.8	8.6	0.01	8.2	8.1	0	9.9	10.3	−0.01
2010	8.3	8.0	0.01	9.1	8.7	0.01	10.7	10.4	0.01
2011	11.2	11.4	−0.01	15.6	15.2	0.01	15.4	14.1	0.03
2012	7.0	6.7	0.01	10.4	10.3	0	11.6	11.9	−0.01
Age group									
20–24	0.1	0.1	0	0.2	0.2	0.01	0.3	0.4	−0.02
25–29	0.2	0.2	0.01	0.3	0.5	−0.02	0.6	0.7	−0.02
30–34	0.6	0.5	0.01	1.3	1.3	0	1.6	1.8	−0.02
35–39	2.4	2.2	0.01	3.1	2.6	0.03	4	4.1	−0.01
40–44	5.9	5.8	0	6	5.6	0.02	7.8	7.8	0
45–49	11.3	11.4	0	10.4	9.9	0.02	12	12.3	−0.01
50–54	15.6	15.5	0	14.9	13.5	0.04	16	14.9	0.03
55–59	15	15.3	−0.01	13.9	14.3	−0.01	14.5	14.3	0.01
60–64	14.6	15	−0.01	14.8	15	−0.01	13.1	13.1	0
65–69	13.4	13.3	0	13.9	14.4	−0.01	11.9	11.9	0
70–74	9.7	9.6	0	10	10.6	−0.02	9	8.8	0.01
75–79	6.2	6.2	0	7	7.2	−0.01	5.3	5.7	−0.01
80–84	3.2	3.4	−0.01	3	3.4	−0.02	2.8	2.8	0
85–89	1.5	1.3	0.01	1.1	1.2	−0.01	0.9	0.9	0
90–94	0.3	0.2	0.01	0.1	0.2	−0.03	0.1	0.2	−0.01
Gender: female	52.5	52.1	0.01	49.5	49.9	−0.01	51.4	51.2	0
Cigarette smoker	5.4	5.3	0	4.9	4.7	0.01	5.4	5.6	−0.01
Medical history									
Acute respiratory disease	56.4	56	0.01	55.9	54.7	0.02	57.9	58.2	−0.01
Chronic liver disease	6.3	6.2	0	9.2	10	−0.03	10.6	11.4	−0.02
Depressive disorder	6.4	6	0.02	7.6	8	−0.02	8.4	8.6	−0.01
Diabetes mellitus	23.6	23.9	−0.01	71.3	81.1	−0.23	31.1	31.7	−0.01
Gastroesophageal reflux disease	7.5	7.1	0.02	8.3	8.5	−0.01	9.5	9.5	0
Hyperlipidemia	32.9	32.6	0.01	46.6	50.2	−0.07	70.1	73	−0.06
Hypertensive disorder	78.7	82.2	−0.09	61.8	64.2	−0.05	66.6	66.7	0
Osteoarthritis	14.9	14.8	0	15.1	14.8	0.01	14.6	14.6	0
Visual system disorder	32.9	32.6	0.01	37.2	37	0	36	35.9	0
Cerebrovascular disease	5.8	5.6	0.01	5.8	6.6	−0.03	7.3	7.2	0
Heart disease	23.1	22.4	0.02	25.5	27.7	−0.05	29.6	30.3	−0.02
Heart failure	5	5	0	5.5	6.3	−0.03	6.4	6.3	0
Ischemic heart disease	9.5	8.7	0.03	14.4	15.2	−0.02	16.3	16.8	−0.01
Peripheral vascular disease	16.6	16.4	0	21.2	21.4	0	18.2	18.8	−0.02
Medication use									
Drugs for *H*. *pylori* eradication	4.3	4.2	0.01	4.7	4.7	0	5.4	5.3	0
Agents acting on the renin‐angiotensin system	38.2	39.7	−0.03	38	39	−0.02	39	39	0
Antibacterials for systemic use	63.8	63.2	0.01	64.4	63.8	0.01	66.5	67	−0.01
Antidepressants	9.8	9.3	0.02	12	11.5	0.02	11.5	11.5	0
Antiepileptics	6	5.7	0.01	7.1	6.9	0.01	7	7.1	0
Anti‐inflammatory and antirheumatic products	56.5	56.1	0.01	56.1	55	0.02	57.1	56.9	0
Antithrombotic agents	42	41.6	0.01	59.3	59.9	−0.01	61.4	62.2	−0.02
Aspirin	NA	NA	NA	30.8	31.9	−0.02	33.0	33.3	−0.01
Beta blocking agents	28.3	28.9	−0.01	23.3	24.2	−0.02	26.3	26.9	−0.01
Calcium channel blockers	54.2	56.3	−0.04	40.1	41.2	−0.02	43.7	44	−0.01
Diuretics	40.4	41.8	−0.03	33.6	33.5	0	35.2	35.3	0
Drugs for acid‐related disorders	60.5	59.7	0.02	61.5	61.7	0	64	64.3	0
Drugs for obstructive airway diseases	35.1	35	0	36.6	36.7	0	36.8	37.3	−0.01
Drugs used in diabetes	18.7	18.9	0	43.2	38.9	0.09	23.6	24.1	−0.01
Metformin	12.7	12.8	0	NA	NA	NA	16.5	16.9	−0.01
Lipid‐modifying agents	21.8	21.4	0.01	32.6	34.2	−0.03	6.9	6.8	0.01
Simvastatin	7.5	7.5	0.00	10.6	11.1	−0.02	NA	NA	NA
Rosuvastatin	1.6	1.5	0.01	3.4	3.5	0.00	NA	NA	NA
Pravastatin	1.4	1.3	0.00	1.9	2.1	−0.02	NA	NA	NA
Pitavastatin	0.8	0.9	0.00	1.5	1.6	0.00	NA	NA	NA
Lovastatin	1.5	1.5	0.00	1.9	2.2	−0.02	NA	NA	NA
Fluvastatin	0.5	0.5	0.01	0.7	0.7	−0.01	NA	NA	NA
Atorvastatin	9.2	9.0	0.01	14.5	15.2	−0.02	NA	NA	NA
Opioids	39.8	39.3	0.01	40.7	40.2	0.01	41.8	41.5	0.01
Psycholeptics	46.1	45.1	0.02	45.5	45.9	−0.01	48.3	48.3	0
Charlson index ‐ Romano adaptation	2.5	2.5	0.02	3.9	3.9	−0.01	3.2	3.2	0

Note

Values are presented as proportion of the patients (%).

Abbreviations: *H*. *pylori*, *Helicobacter pylori*; NA, not applicable; SMD, standard mean difference.

### 3.1 Aspirin

The median exposure duration for aspirin in the target cohort was 2.4 years (interquartile range [IQR] 1.5–4.1 years). The median period from cohort entry to gastric cancer diagnosis was 4.2 years.

**FIGURE 1:**
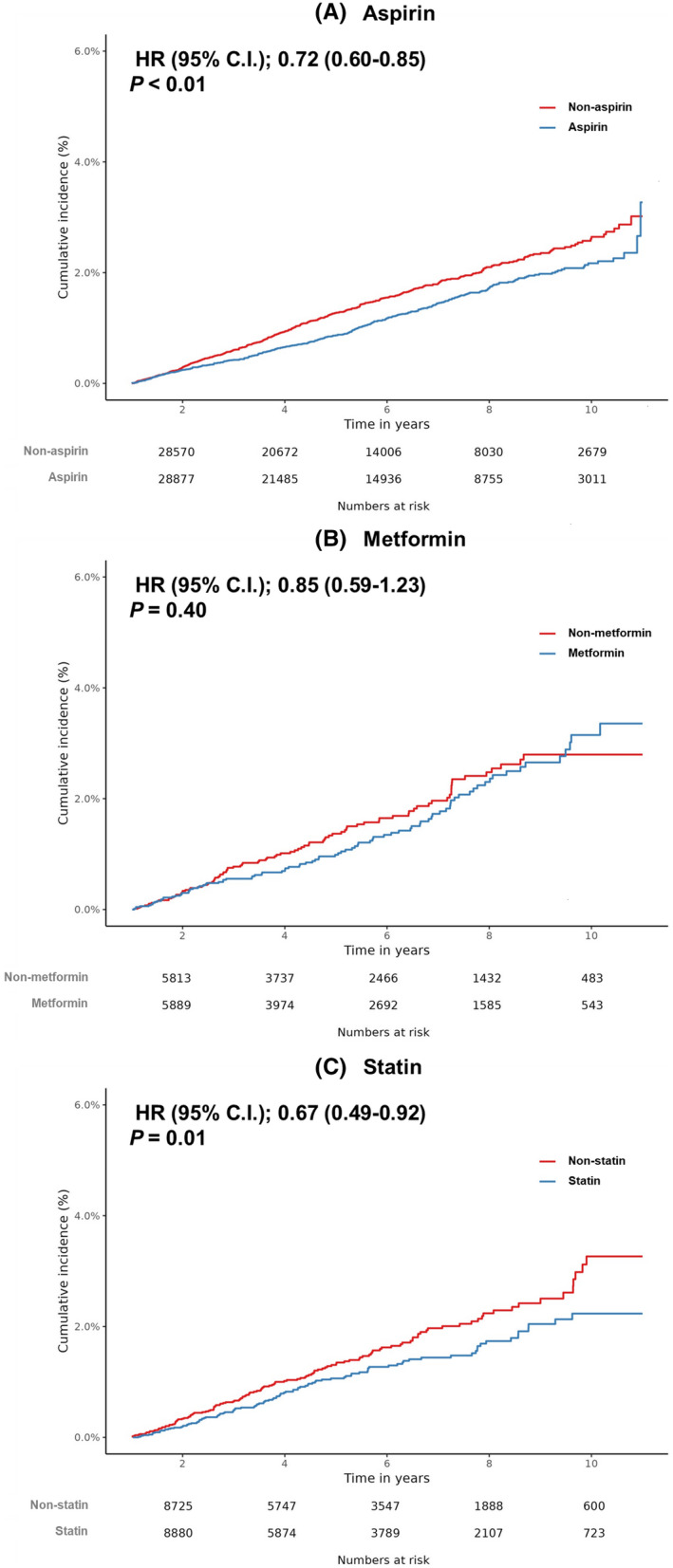
Kaplan–Meier curve for cumulative incidence of gastric cancer between the target and comparator cohorts in the analysis of aspirin (A), metformin (B), and statin (C)

The Kaplan–Meier curve showed the cumulative incidence of gastric cancer in the target and comparator cohorts (Figure 1A). During the median 4.7‐year observation period, 369 and 450 subjects in the target and comparator cohorts developed gastric cancer, respectively. The risk of gastric cancer was lower in the target cohort compared to that in the comparator cohort (HR [95% CI] = 0.72 [0.60–0.85], *p* < 0.01) (Table 2). The results of sensitivity analysis according to different matching ratios and lag periods are shown in Table 3. The analysis under 1:4 matching with a 6‐month lag period also showed the significant consistent result with main analysis, although that under a longer lag period (1 year) failed to show a significant result. The analysis in the DM population showed that aspirin tended to be associated with a lower risk of gastric cancer development although statistical significance was not identified (HR [95% CI] = 0.79 [0.60–1.03], *p *= 0.08) (Table 4).

**TABLE 2:** Impacts of aspirin, metformin, and statin on the risk of gastric cancer development

Target drug	Cohort	Subjects, *n*	Observation, person‐years	Gastric cancer development, *n*	Incidence rate, /1000 person‐years	HR (95% CI)	*p*‐value
Aspirin	Target	31,839	154,136	369	2.39	0.72 (0.60–0.85)	<0.01
	Comparator	31,839	148,104	450	3.04	Reference	
Metformin	Target	6764	29,358	91	3.10	0.85 (0.59–1.23)	0.40
	Comparator	6764	27,944	94	3.36	Reference	
Statin	Target	10,251	42,659	106	2.48	0.67 (0.49–0.92)	0.01
	Comparator	10,251	41,288	136	3.29	Reference	

Abbreviations: CI, confidence interval; HR, hazard ratio.

**TABLE 3:** Sensitivity analyses according to matching ratios and lag periods

Propensity score matching ratio (target cohort: comparator cohort)	Lag period	Aspirin, HR (95% CI), *p*‐value	Metformin, HR (95% CI), *p*‐value	Statin, HR (95% CI), *p*‐value
1: 1 (main analysis)	1 year	0.72 (0.60–0.85), <0.01	0.85 (0.59–1.23), 0.40	0.67 (0.49–0.92), 0.01
1: 4	1 year	0.71 (0.61–0.83), <0.01	1.01 (0.72–1.41), 0.97	0.69 (0.52–0.91), 0.01
1: 1	2 year	0.81 (0.67–0.98), 0.03	1.29 (0.83–2.01), 0.27	0.68 (0.47–0.97), 0.04
1: 4	2 year	0.79 (0.67–0.94), 0.01	1.24 (0.84–1.83), 0.28	0.70 (0.50–0.96), 0.03

Abbreviations: CI, confidence interval; HR, hazard ratio.

**TABLE 4:** Impacts of aspirin, metformin, and statin on the risks of gastric cancer development in patients with diabetes mellitus

Target drug	Cohort	Subjects, *n*	Observation, person‐years	Gastric cancer development, *n*	Incidence rate, /1000 person‐years	HR (95% CI)	*p*‐value
Aspirin	Target	11,216	55,922	179	3.20	0.79 (0.60–1.03)	0.08
	Comparator	11,216	52,678	186	3.53	Reference	
Metformin	Target	5566	29,019	113	3.89	0.98 (0.70–1.39)	0.93
	Comparator	5566	25,895	96	3.71	Reference	
Statin	Target	6530	31,018	83	2.68	0.60 (0.43–0.85)	<0.01
	Comparator	6530	28,745	125	4.35	Reference	

Abbreviations: CI, confidence interval; HR, hazard ratio.

### 3.2 Metformin

The median exposure duration for metformin in the target cohort was 2.3 years (IQR 1.5–3.7 years). The median period from cohort entry to gastric cancer diagnosis was 4.5 years. Figure 1B shows the Kaplan–Meier curve for the cumulative incidence of gastric cancer. During the median 4.2‐year observation period, 91 and 94 subjects in the target and comparator cohorts were diagnosed with gastric cancer, respectively. The Cox proportional hazard model did not identify a preventive effect of metformin against gastric cancer (metformin ≥365 days; HR [95% CI] = 0.85 [0.59–1.23]; *p* = 0.40) (Table 2). As shown in Table 3, sensitivity analyses showed no significant difference in the risks of gastric cancer development in the target and comparator cohorts. Additionally, no significant impact of metformin on the risk of gastric cancer development was observed in the DM population (HR [95% CI] = 0.98 [0.70–1.39], *p *= 0.93) (Table 4).

### 3.3 Statin

The median exposure duration for statin in the target cohort was 2.0 years (IQR 1.4–0.3 years). The median period from cohort entry to gastric cancer diagnosis was 3.6 years. The Kaplan–Meier curve showed higher cumulative incidence of gastric cancer in the comparator cohort compared to that in the target cohort (Figure 1C). During the median 4.1‐year observation period, 106 and 136 subjects, respectively, in the target and comparator cohorts developed gastric cancer. The risk of gastric cancer development in the target cohort was lower than that in the comparator cohort (statin ≥365 days; HR [95% CI] = 0.67 [0.49–0.92]; *p *= 0.01) (Table 2). The impact of statin on the risk of gastric cancer was also shown in the results of the sensitivity analyses (Table 3). Statin use was also associated with a lower risk of gastric cancer development in the DM population (HR [95% CI] = 0.60 [0.43–0.85], *p *< 0.01) (Table 4).

### 3.4 Systematic review and meta‐analysis of previous studies

The meta‐analysis included 18, 9, and 11 studies on aspirin, metformin, and statin, respectively (Figure S5).^4^, ^5^, ^6^, ^7^, ^8^, ^9^, ^10^, ^22^, ^23^, ^24^, ^25^, ^26^, ^27^, ^28^, ^29^, ^30^, ^31^, ^32^, ^33^, ^34^, ^35^, ^36^, ^37^, ^38^, ^39^, ^40^, ^41^, ^42^, ^43^, ^44^, ^45^, ^46^, ^47^, ^48^, ^49^, ^50^ The characteristics of the included studies are summarized in Tables S4–S6.

**FIGURE 2:**
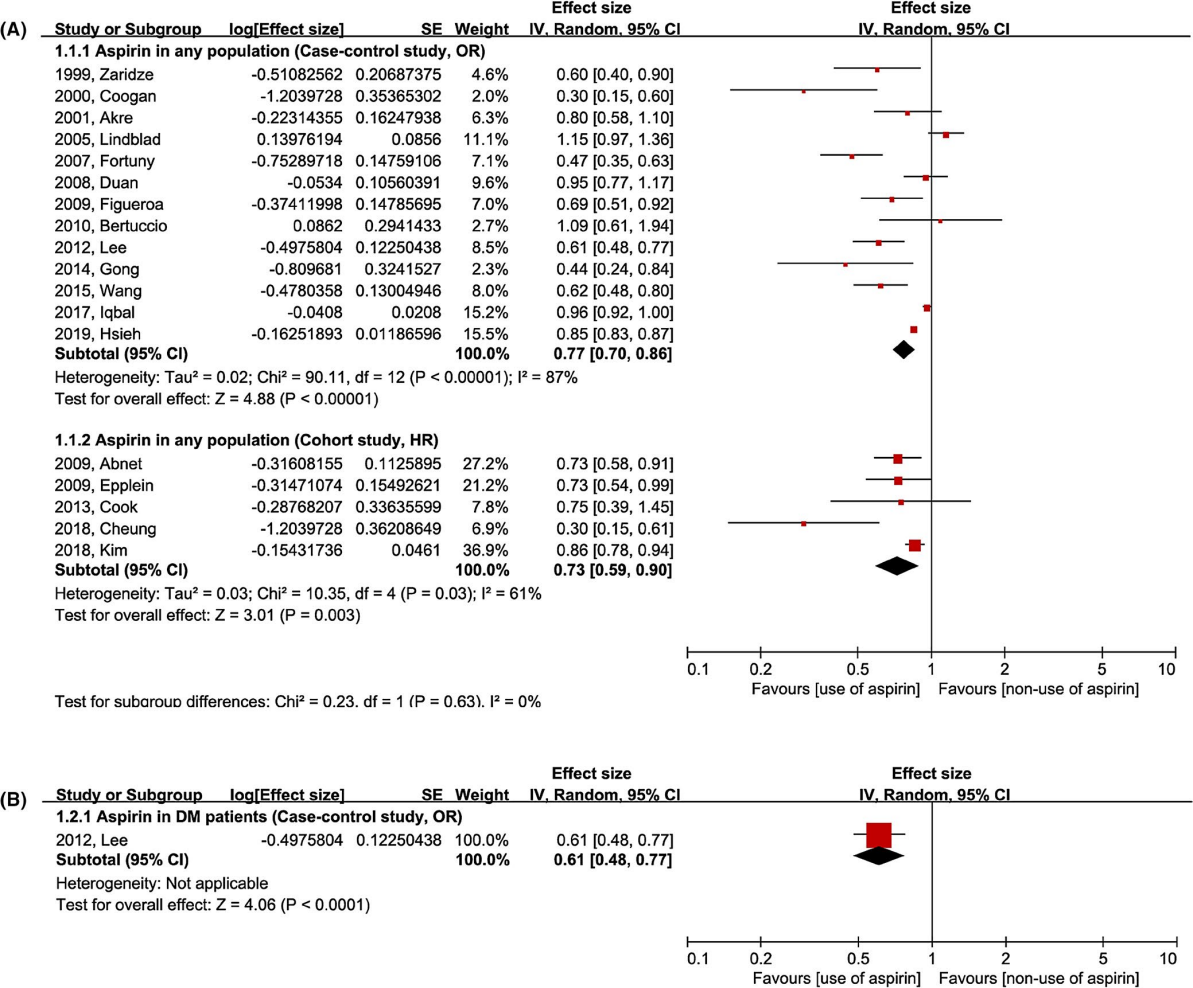
Meta‐analysis of gastric cancer prevention by aspirin. (A) Any population, (B) DM population. CI, confidence interval; DM, diabetes mellitus; HR, hazard ratio; IV, inverse variance; OR, odds ratio; SE, standard error

The risk of gastric cancer development according to the use of aspirin is evaluated in 13 case–control and 5 cohort studies. The use of aspirin showed a lower risk of gastric cancer development compared to the non‐use of aspirin in both case–control and cohort studies (case–control: OR [95% CI] = 0.77 [0.70–0.86]; cohort: HR [95% CI] = 0.73 [0.59–0.90]; Figure 2). Significant heterogeneity was identified in both study design subgroups (case–control: df = 12, *p* < 0.01, *I* ^2^ = 87%; cohort: df = 4, *p* = 0.03, *I* ^2^ = 61%). Although only one study investigated the risk of gastric cancer development in patients with DM who took aspirin, the use of aspirin was beneficial to prevent gastric cancer development in this population (Figure 2B).^31^

**FIGURE 3:**
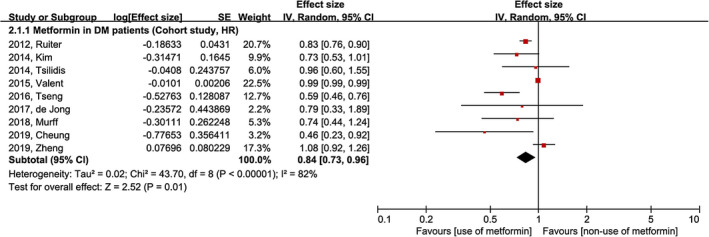
Meta‐analysis of gastric cancer prevention by metformin in the DM population. CI, confidence interval; DM, diabetes mellitus; HR, hazard ratio; IV, inverse variance; SE, standard error

All nine included studies on the risk of gastric cancer development according to the use of metformin included only DM patients and were designed as cohort studies. Although significant heterogeneity was observed, the risk of gastric cancer development for the use of metformin was lower than that in for the non‐use of metformin (HR [95% CI] = 0.84 [0.73–0.96], df = 8, *p* < 0.01, *I* ^2^ = 82%; Figure 3).

**FIGURE 4:**
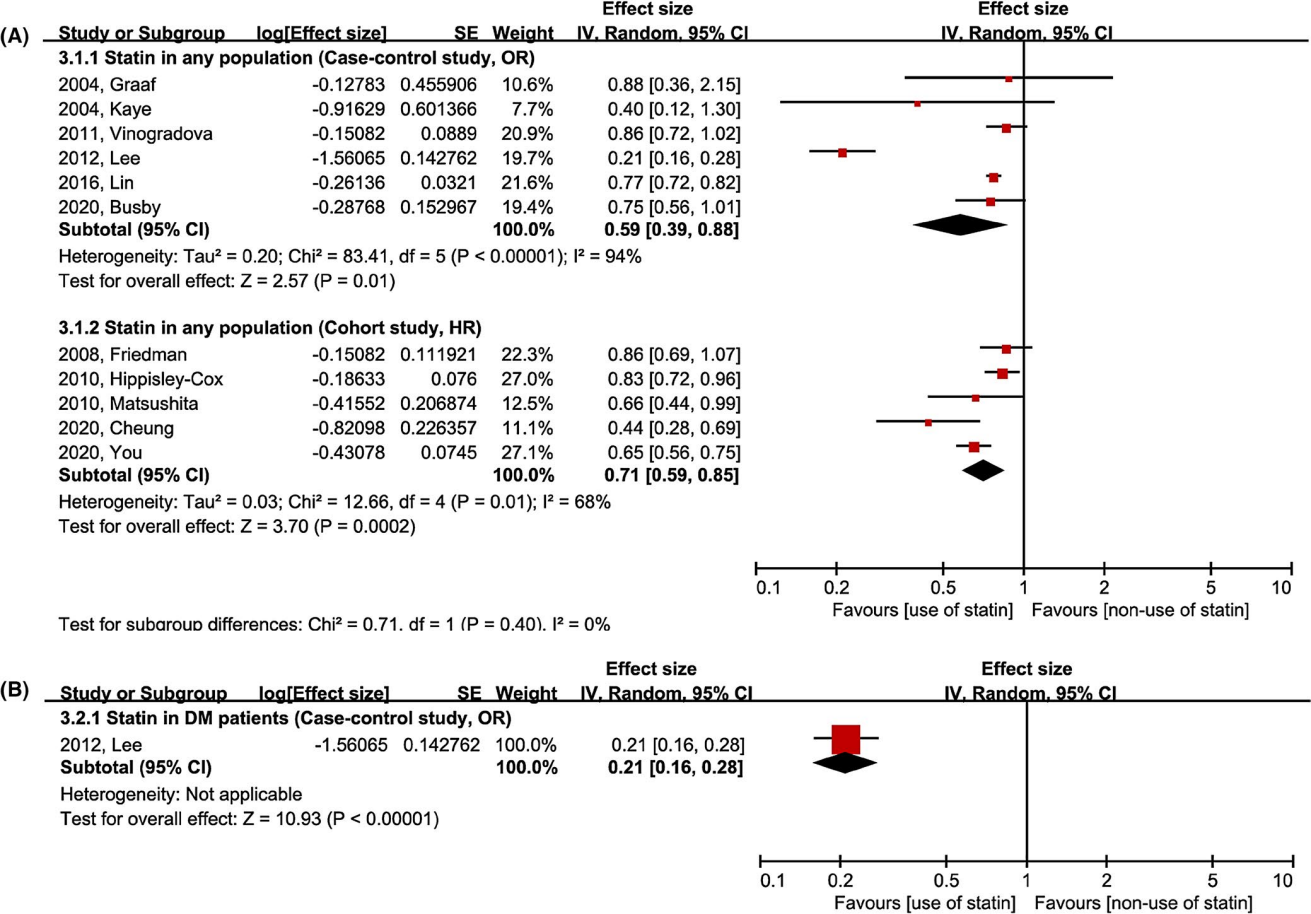
Meta‐analysis of gastric cancer prevention by statin. (A) Any population, (B) DM population. CI, confidence interval; DM, diabetes mellitus; HR, hazard ratio; IV, inverse variance; OR, odds ratio; SE, standard error

Figure 4 shows the comparative risk of gastric cancer development between the use and non‐use of statin groups. In the six case–control studies, the pooled OR of statin use was 0.59 (95% CI, 0.39–0.88), while the pooled HR of statin use was 0.71 (95% CI, 0.59–0.85) in the five cohort studies. Significant heterogeneity was identified in both case–control and cohort studies. The study by Lee et al. that included only patients with DM also demonstrated that the use of statin lowered the risk of gastric cancer development (Figure 4B).^31^

The forest plots of subgroup analysis according to study regions (Eastern vs. Western studies) are shown in Figures [Link], [Link], [Link]. The effect size of aspirin for the risk of gastric cancer development did not differ between Eastern and Western studies (case–control: Eastern studies, OR 0.79 [95% CI 0.70–0.89]; Western studies, OR 0.73 [95% CI 0.56–0.95]; *p* = 0.59, *I* ^2^ = 0%; cohort: Eastern studies, HR 0.54 [95% CI 0.19–1.50]; Western studies, HR 0.73 [95% CI 0.62–0.87]; *p* > 0.99, *I* ^2^ = 0%). However, the effect size of metformin or statin for the risk of gastric cancer development in cohort studies was lower in Eastern studies compared to Western studies (metformin: Eastern studies, HR 0.62 [95% CI 0.52–0.75]; Western studies, HR 0.94 [95% CI 0.83–1.06]; *p* < 0.01, *I* ^2^ = 92.2%; statin: Eastern studies, HR 0.61 [95% CI 0.50–0.74]; Western studies, HR 0.84 [95% CI 0.74–0.95]; *p* < 0.01, *I* ^2^ = 86%).

## 4 DISCUSSION

This was the first Korean nationwide population‐based study to evaluate the impact of aspirin, metformin, and statin on gastric cancer incidence using OMOP‐CDM‐based analysis. In the present study, statin use over 1 year significantly reduced gastric cancer incidence by 33% and a protective effect was also identified in the DM subgroup population. To date, few studies have assessed the risk of gastric cancer with statin use, especially in Asia. Our findings enhanced the understanding of the additional role of statins as chemopreventive agents in the gastric cancer.

Aspirin use over 1 year exhibited a protective effect against gastric cancer development by 28%, whereas metformin was not associated with gastric cancer. Our results of aspirin and statin use were generally consistent with those of the systematic review and meta‐analysis, although these results should be interpreted with caution that significant heterogeneity was observed in the meta‐analyses. In the sensitivity analyses, the chemopreventive effect with 2‐year lag period and 1:4 matching was also significant.

In the present study, no association was observed between metformin use and the risk of gastric cancer development in both general and DM populations. This finding was contrary to the result of the meta‐analysis, which showed a significant risk reduction in patients using metformin. However, the results of meta‐analysis require cautious interpretation owing to the significant heterogeneity. Among the nine studies included in the meta‐analysis of metformin use, five showed insignificant outcomes although the pooled effect size was significant. Importantly, the impact of metformin on gastric cancer development differed according to the study regions (Eastern vs. Western countries) as shown in our subgroup meta‐analysis. Furthermore, one Korean study included in the meta‐analysis for metformin did not show a significant reduction of the risk of gastric cancer by metformin.^38^ More investigations with different ethnic populations may be needed to clarify the chemopreventive effect of metformin against gastric cancer.

Our study has several strengths. First, to reduce immortal time bias, we used a new‐user study design to all analyses. Immortal time bias may be a concern in observational studies since the addition of immortal person‐time to a given treatment group leads to an underestimation of the true risk in that group and spurious beneficial effects of treatments.^51^, ^52^, ^53^, ^54^ We confined the study cohort to subjects who were exposed to target drugs for the first time and 1 year of observation period before the index date. Moreover, we further analyzed the associations between the use of target drugs and the risk of gastric cancer in a population with DM. These analyses compared patients treated with the target drugs to those treated with oral hypoglycemic agents without target drugs. Second, it was based on a longitudinal Korean nationwide cohort in a region in which gastric cancer is prevalent. To our knowledge, there was no study evaluating the impacts of three different drugs under the same analytic methods and database in high‐risk region of gastric cancer, whereas the previous studies applied heterogeneous analytic methods and covariates. Third, we used a database converted by OMOP‐CDM. Because OHDSI research use standardized vocabularies, which can be applied to other OMOP‐CDM‐based databases worldwide. In addition, we conducted a large‐scale propensity score matching analysis, which includes all drugs, diagnosis, and disease severity as covariates. The large‐scale propensity score matching was used to make the two cohorts more comparable to each other, and most of SMD after propensity matching was lower than 0.1. To reduce the residual bias, we performed the analysis of negative control outcomes. Lastly, the results of multiple sensitivity analyses with varying lag periods and matching ratios showed the robustness of the study findings. Additionally, the systematic review and meta‐analyses helped to better understand and validate our study findings.

This study also has some potential limitations. First, although *H*. *pylori* infection is the strongest known risk factor of gastric cancer, we could not identify infection status in NHIS‐CDM database. To minimize the bias caused by differences in *H*. *pylori* infection status, we adjusted for drugs used in *H*. *pylori* eradication therapy before the index date between the target and comparator cohorts. Previous studies that included only patients who received *H*. *pylori* eradication therapy showed that aspirin, metformin, and statin use were associated with decreased risk of gastric cancer after *H*. *pylori* eradication.^4^, ^10^, ^43^ Second, histopathologic findings including intestinal metaplasia and histologic type of gastric cancer were unavailable in our database. Niikura et al. recently demonstrated that long‐term (>2 years) aspirin use revealed preventive effects of gastric cancer only in diffuse‐type cancer.^55^ Thus, the chemopreventive effects of drugs might depend on the histologic type of cancer.^55^ Third, we included subjects taking both aspirin and statin in the analysis, which are often prescribed simultaneously in cardiac disease. It may lead to exaggerated result. However, exclusion of users with multiple drugs may not reflect the real‐world practice, hence, we adjusted all previous drugs before cohort entry. Fourth, in spite of our maximal efforts to reduce the possible biases, our study was observational study, therefore, we could not demonstrate causal relationship. The golden standard of study design is randomized controlled trial (RCT) to reveal causal relation between chemopreventive drugs and gastric cancer prevention. But, there are many difficulties such as long‐term follow‐up periods, huge research cost, and ethic problem to conduct RCT because the end point of this study is cancer occurrence. Fifth, the OMOP‐CDM converted database has limitations from data conversion process. There might be misclassification bias from erroneous data conversion.

Despite the limitations, our study provides a comprehensive understanding of the impact of aspirin, metformin, and statin use on the risk of gastric cancer development. Statin or aspirin use was associated with a lower risk of gastric cancer development, while the beneficial effect of metformin in gastric cancer prevention was not observed. The protective effect of statin against gastric cancer was also significant in patients with diabetes mellitus.

## ETHICS APPROVAL STATEMENT

The protocol of the current study was approved by the Institutional Review Board of Kangdong Sacred Heart Hospital (IRB no. 2019‐05‐014).

## CONFLICT OF INTERESTS

None.

## AUTHOR CONTRIBUTIONS

SIS and CHP; study concept and design, acquisition of data, analysis and interpretation of data, and drafting of the manuscript. TJK and CSB; critical revision of the manuscript for important intellectual content. JYK and KJL; acquisition of data; analysis and interpretation of data. HHK, JK, and SCY; statistical analysis, technical, or material support. WGS; study concept and design, obtained funding, and study supervision.

## Supporting information

Fig S1

Fig S2

Fig S3

Fig S4

Fig S5

Fig S6

Fig S7

Fig S8

Table S1‐S6

Appendix S1

## Data Availability

Data are available in the website https://github.com/rlawodud3920.
