# Supplementary figures and images for: Aspirin, metformin, and statin use on the risk of gastric cancer: A nationwide population‐based cohort study in Korea with systematic review and meta‐analysis
Source: Cancer Med. 2021 Dec 30;11(4):1217–31. doi: 10.1002/cam4.4514 (PMC8855895; doi:10.1002/cam4.4514)

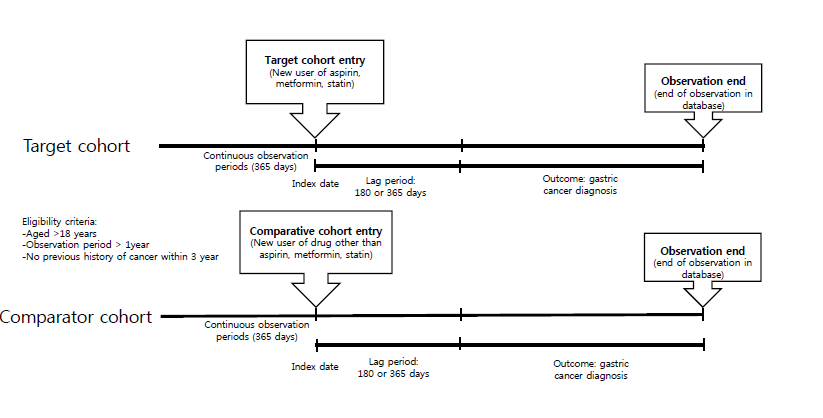

Supplement: Supplementary file 1 — Fig S1 [file CAM4-11-1217-s004.tif]

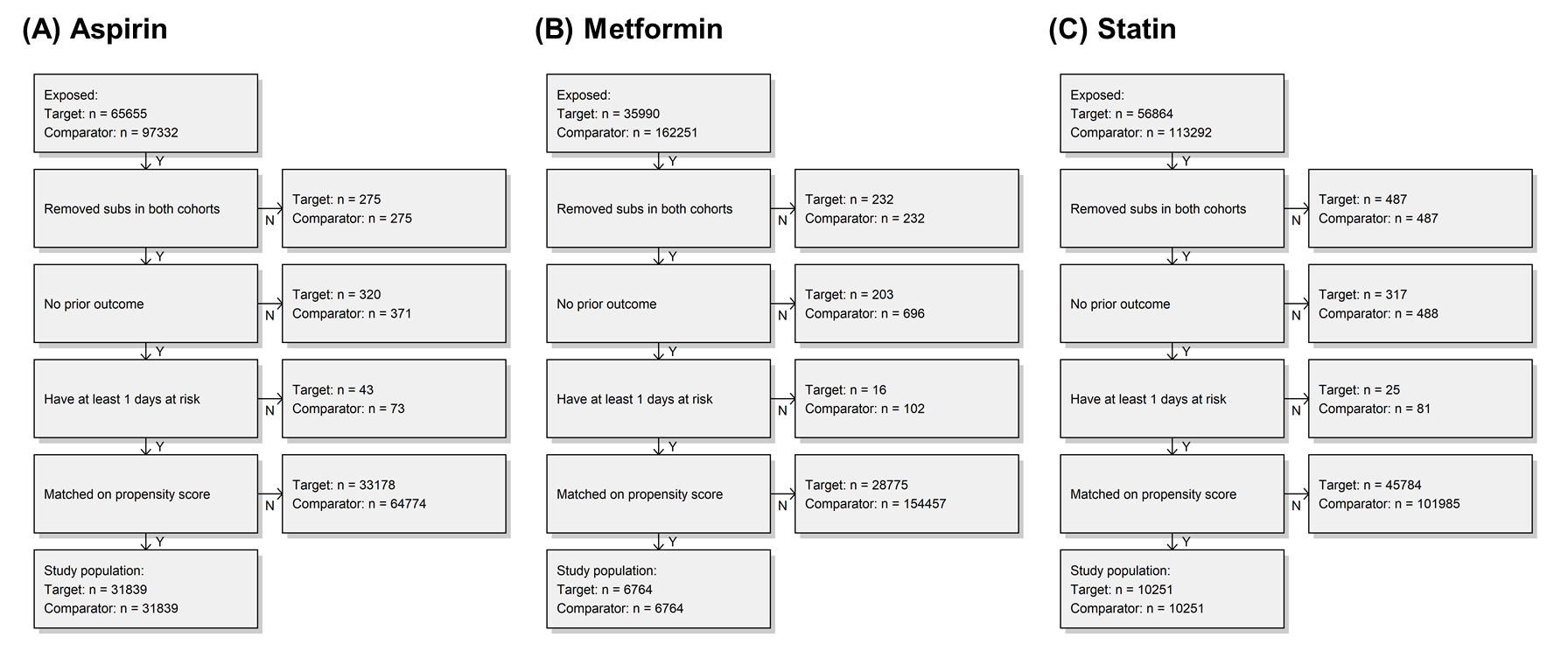

Supplement: Supplementary file 2 — Fig S2 [file CAM4-11-1217-s010.tif]

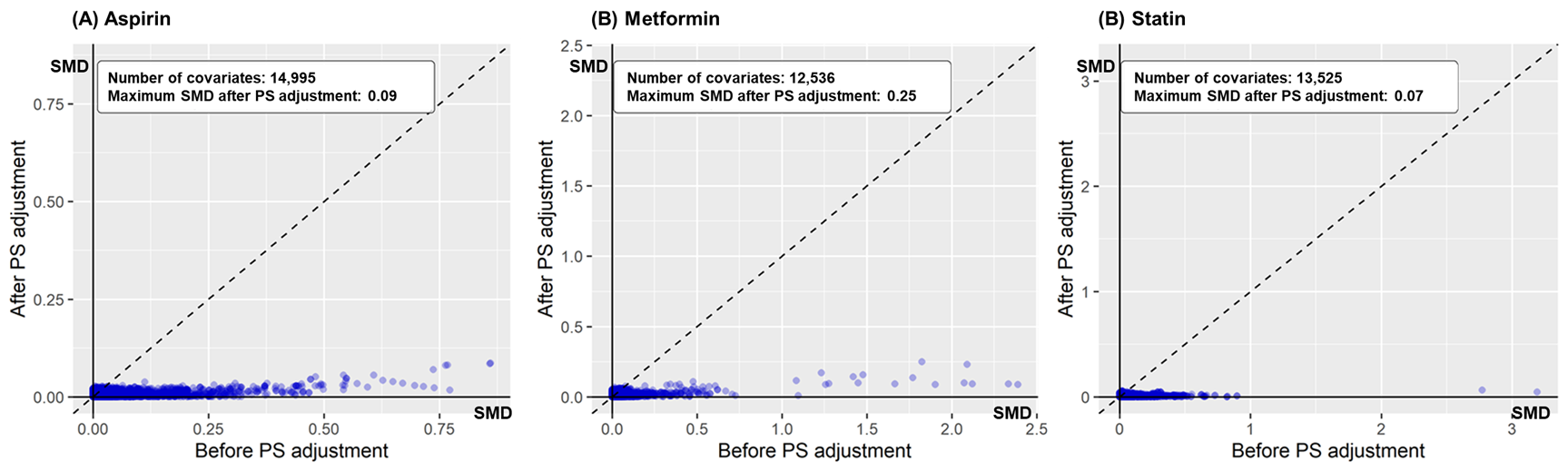

Supplement: Supplementary file 3 — Fig S3 [file CAM4-11-1217-s006.tif]

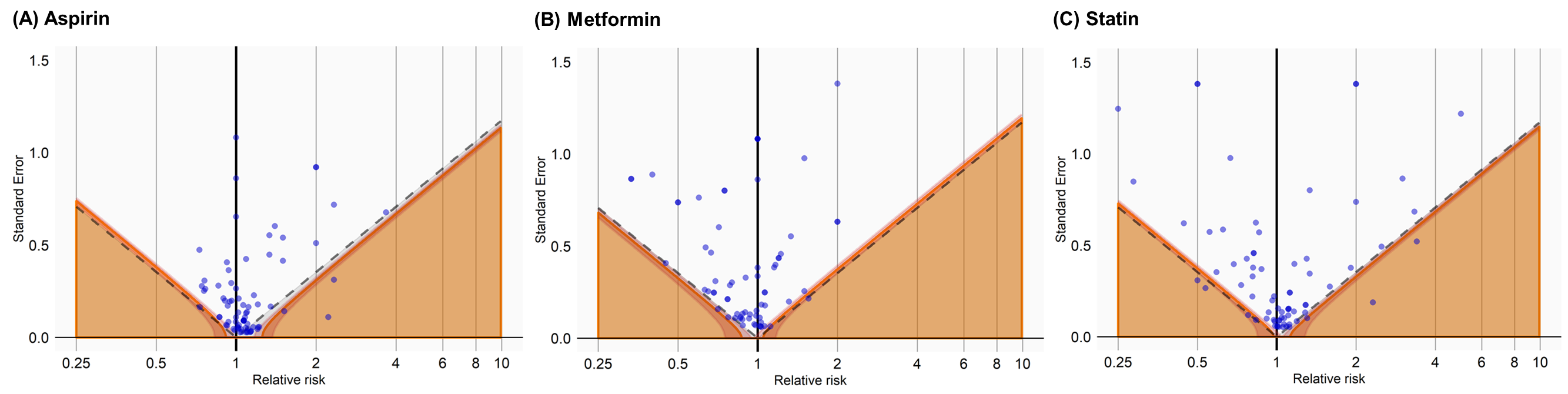

Supplement: Supplementary file 4 — Fig S4 [file CAM4-11-1217-s005.tif]

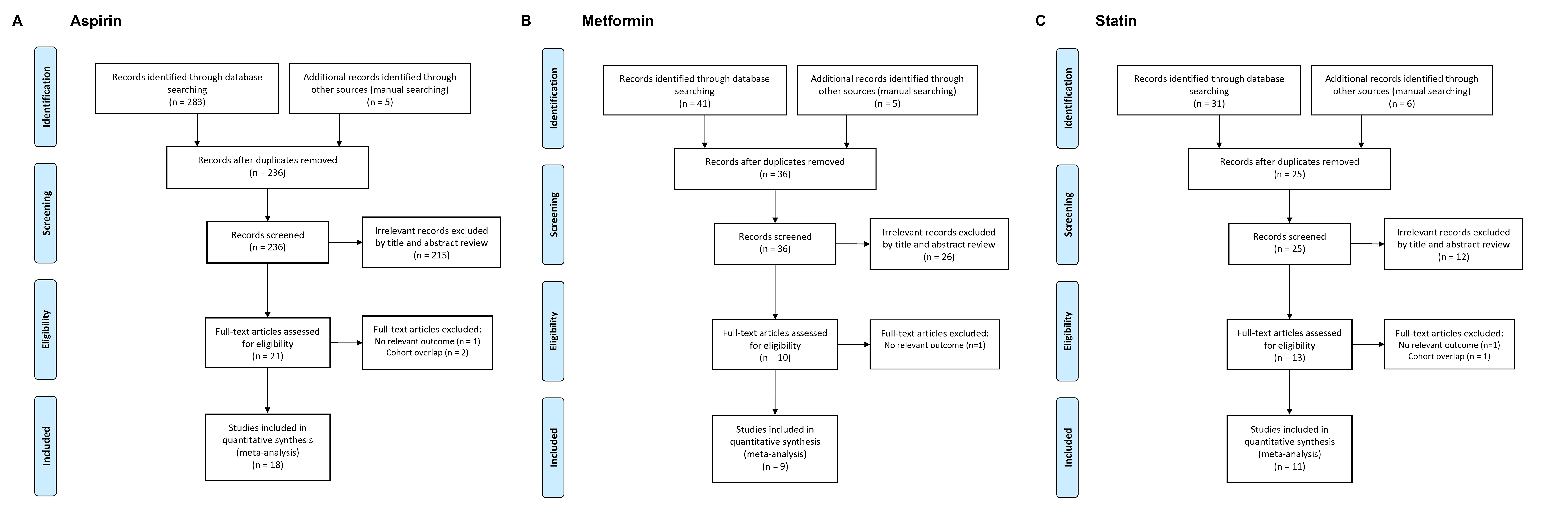

Supplement: Supplementary file 5 — Fig S5 [file CAM4-11-1217-s007.tif]

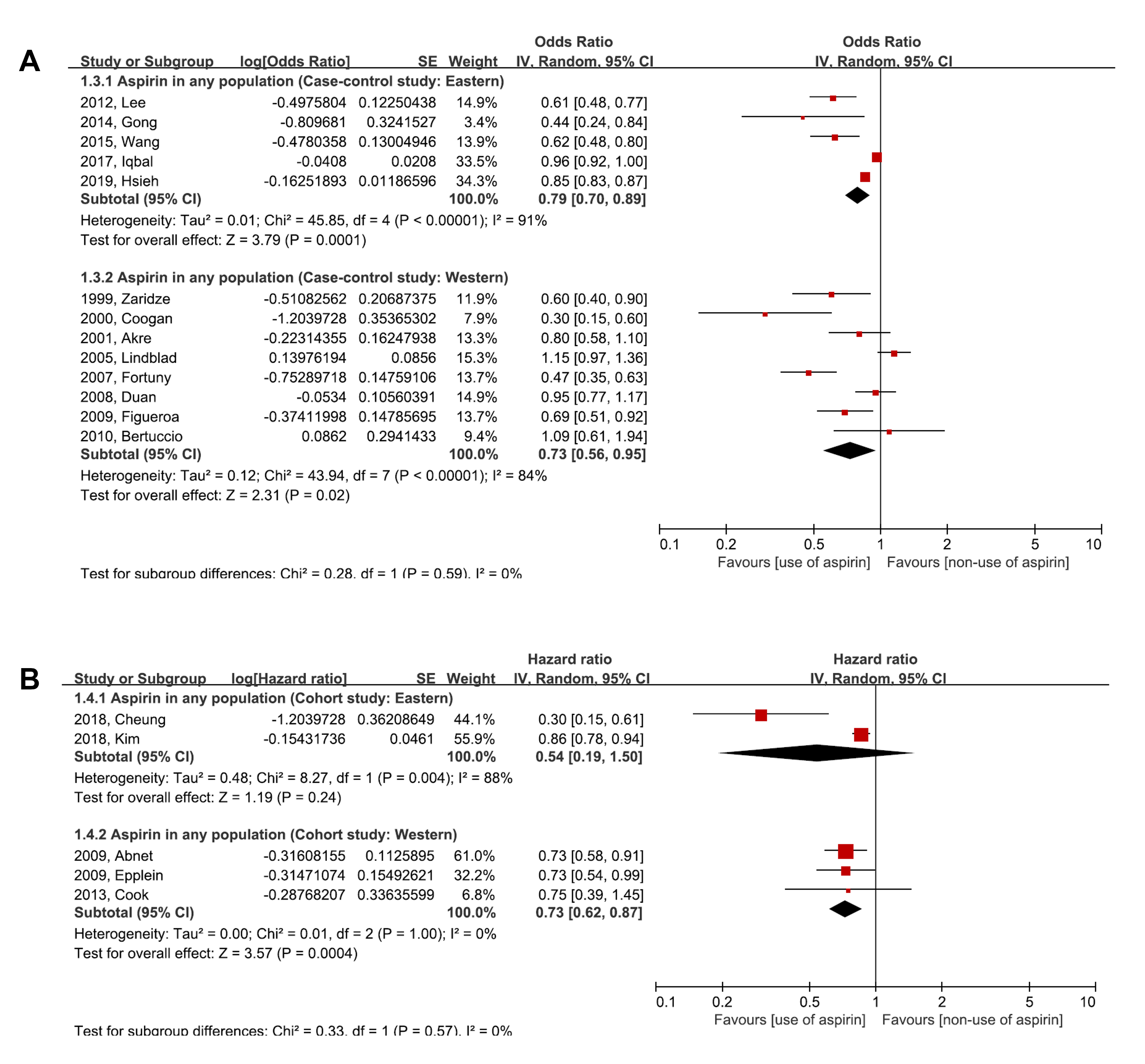

Supplement: Supplementary file 6 — Fig S6 [file CAM4-11-1217-s001.tif]

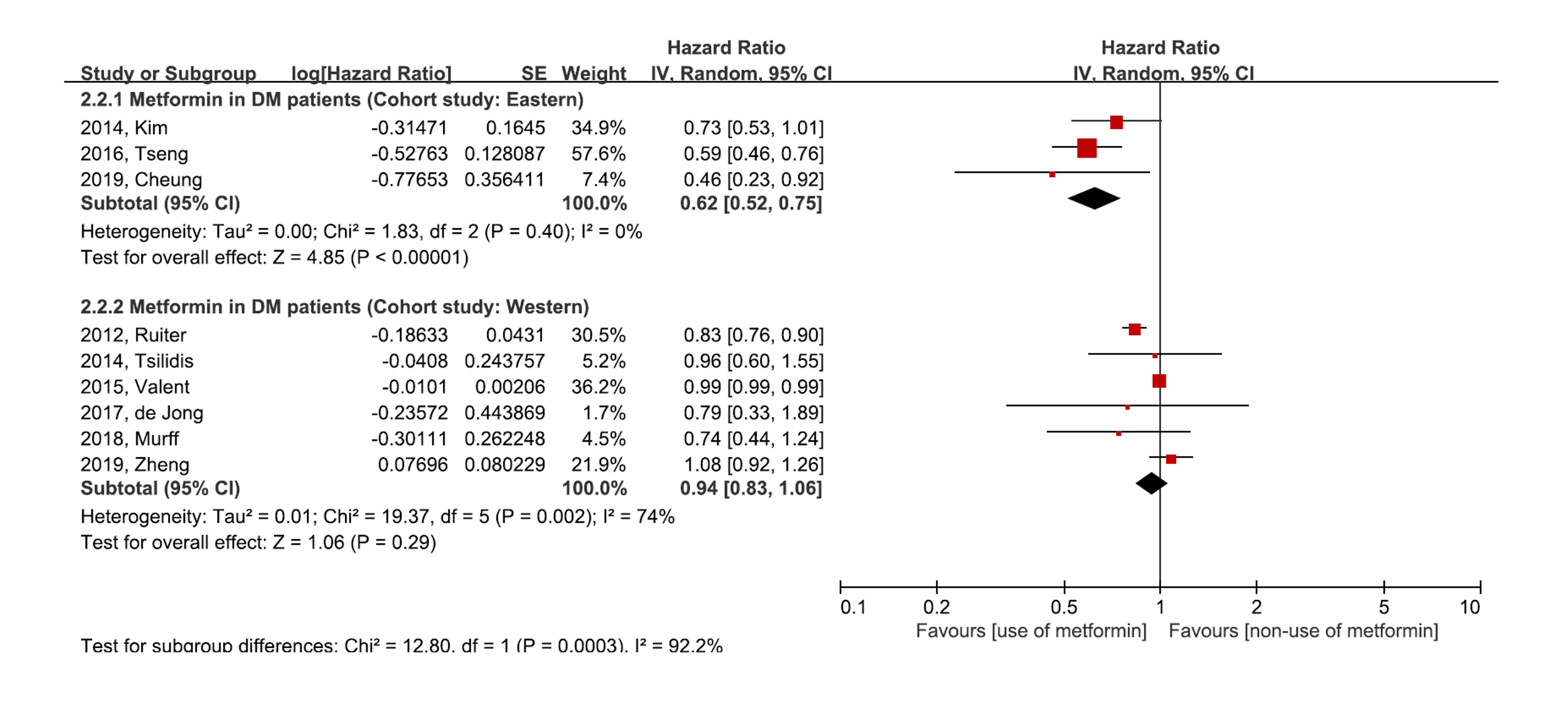

Supplement: Supplementary file 7 — Fig S7 [file CAM4-11-1217-s008.tif]

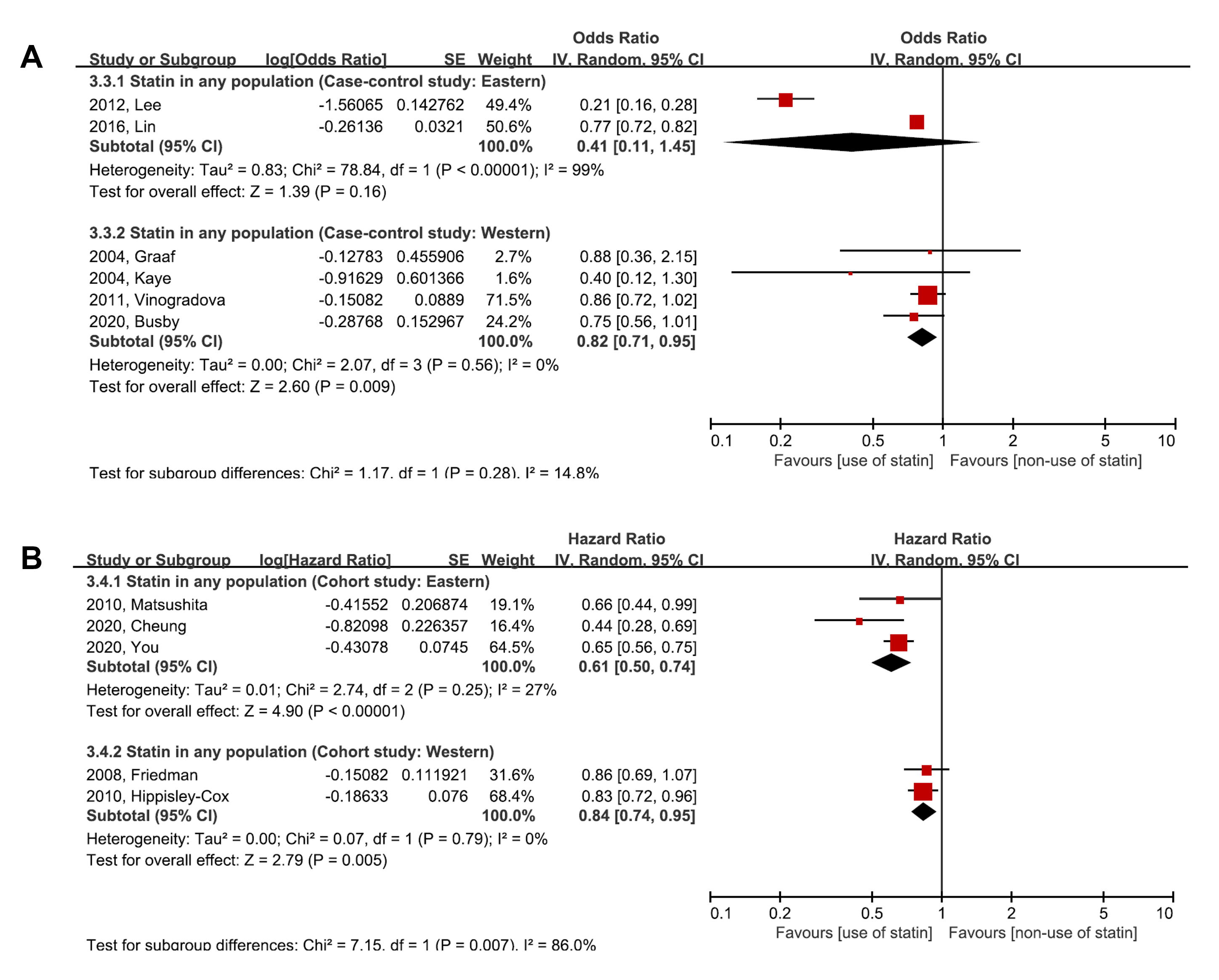

Supplement: Supplementary file 8 — Fig S8 [file CAM4-11-1217-s009.tif]
