# Supplementary material for: Aspirin, metformin, and statin use on the risk of gastric cancer: A nationwide population‐based cohort study in Korea with systematic review and meta‐analysis
Source: Cancer Med. 2021 Dec 30;11(4):1217–31. doi: 10.1002/cam4.4514 (PMC8855895; doi:10.1002/cam4.4514)
Supplement: Supplementary file 9 — Table S1‐S6 [file CAM4-11-1217-s003.docx]

| **Class** | **Concept IDs** |
| --- | --- |
| Aspirin | Aspirin (1112807) |
| Metformin | Metformin (1503297) |
| Statin | Simvastatin (1539403); rosuvastatin (1510813); Pravastatin (1551860); pitavastatin (40165636); lovastatin (1592085); fluvastatin (1549686); atorvastatin (1545958) |
| PPI | Rabeprazole (911735); pantoprazole (948078); omeprazole (923645); lansoprazole (929887); esomeprazole (904453); dexlansoprazole (19039926) |
| Amoxicillin | Amoxicillin (1713332); amoxicillin 500 mg oral capsule (19073187) |
| Clarithromycin | Clarithromycin (1750500); clarithromycin 500 mg oral tablet (Klaricid) (21113674); |
| Bismuth | Bismuth subcitrate (958134) |
| Metronidazole | Metronidazole 250 mg oral tablet (19080187); metronidazole (1707164) |
| Tetracycline | Tetracycline oral capsule (40087792); tetracycline 250 mg oral capsule (19019852) |
| Oral hypoglycemic agents | Acarbose (1529331); acetohexamide (1530014); carbutamide (19033498); chlorpropamide (1594973); gliclazide (19059796); insulin glargine (1502905); insulin lispro (1550023); insulin, aspart, human (1567198); insulin, glulisine, human (1544838); insulin, regular, pork (1586346); lixisenatide (44506754); phenformin (19033909); regular insulin, human (1596977); tolazamide (1502809); tolbutamide (1502855); vanadium (19007693); alogliptin (43013884); canagliflozin (43526465); dapagliflozin (44785829); dulaglutide (45774435); empagliflozin (45774751); exenatide (1583722); glibornuride (19001409); glimepiride (1597756); gliquidone (19097821); insulin degludec (35602717); insulin detemir (1516976); insulin, isophane (46221581); liraglutide (40170911); miglitol (1510202); nateglinide (1502826); pioglitazone (1525215); repaglinide (1516766); rosiglitazone (1547504); saxagliptin (40166035); sitagliptin (1580747); troglitazone (1515249); vildagliptin (19122137) |

**Table S1. OMOP-CDM drug codes included in the current study**

OMOP-CDM, Observational Medical Outcomes Partnership Common Data Model

**Table S2. List of negative control outcomes**

| **Concept ID** | **Concept Code** | **Concept Name** |
| --- | --- | --- |
| 378256 | 46670006 | Abnormal reflex |
| 440424 | 87486003 | Aphasia |
| 439237 | 52684005 | Assault |
| 378424 | 82649003 | Astigmatism |
| 261880 | 46621007 | Atelectasis |
| 134118 | 400190005 | Atrophic condition of skin |
| 4224118 | 40492006 | Bladder dysfunction |
| 80509 | 203465002 | Bone cyst |
| 434626 | 20010003 | Borderline personality disorder |
| 438407 | 78004001 | Bulimia nervosa |
| 134765 | 238108007 | Cachexia |
| 4172458 | 49883006 | Candidiasis of skin |
| 436740 | 17382005 | Cervical incompetence |
| 381581 | 1482004 | Chalazion |
| 4307254 | 423125000 | Closed fracture |
| 4047787 | 123971006 | Colles' fracture |
| 198075 | 240542006 | Condyloma acuminatum |
| 73302 | 64217002 | Curvature of spine |
| 4242416 | 58588007 | Cutis laxa |
| 433163 | 238107002 | Deficiency of macronutrients |
| 4047269 | 229844004 | Deformity of foot |
| 133228 | 80967001 | Dental caries |
| 4147672 | 30415006 | Disease due to Papilloma virus |
| 4153380 | 371160000 | Disorder of carotid artery |
| 4140510 | 3305006 | Disorder of lymphatic vessel |
| 433440 | 78667006 | Dysthymia |
| 376132 | 62909004 | Ectropion |
| 440695 | 302690004 | Encopresis |
| 438872 | 267023007 | Excessive eating - polyphagia |
| 78804 | 27431007 | Fibrocystic disease of breast |
| 4131595 | 12676007 | Fracture of radius |
| 74855 | 33839006 | Genital herpes simplex |
| 441788 | 240532009 | Human papilloma virus infection |
| 76737 | 55434001 | Hydrocele |
| 4029582 | 237793004 | Hyperandrogenization syndrome |
| 195212 | 47270006 | Hypercortisolism |
| 438134 | 77692006 | Hypersomnia |
| 140362 | 36976004 | Hypoparathyroidism |
| 4322737 | 427898007 | Infection of tooth |
| 4207688 | 55184003 | Infectious enteritis |
| 79072 | 266579006 | Inflammatory disorder of breast |
| 139099 | 400097005 | Ingrowing nail |
| 4288544 | 396232000 | Inguinal hernia |
| 444191 | 125593007 | Injury of face |
| 444130 | 125604000 | Injury of foot |
| 134222 | 125597008 | Injury of forearm |
| 4029966 | 128609009 | Intracranial aneurysm |
| 437409 | 127296001 | Intracranial injury |
| 4297984 | 76844004 | Local infection of wound |
| 4018050 | 10443009 | Localized infection |
| 439840 | 1415005 | Lymphangitis |
| 4163232 | 45198002 | Mastitis |
| 440389 | 91138005 | Mental retardation |
| 436100 | 60380001 | Narcolepsy |
| 4262178 | 397732007 | Neurogenic dysfunction of the urinary bladder |
| 193874 | 8009008 | Nocturnal enuresis |
| 4171549 | 419153005 | Nodular goiter |
| 442274 | 52073004 | Oligomenorrhea |
| 4215978 | 414941008 | Onychomycosis |
| 4171915 | 274718005 | Orchitis |
| 380731 | 3135009 | Otitis externa |
| 378160 | 65668001 | Otorrhea |
| 192606 | 60389000 | Paraplegia |
| 253796 | 36118008 | Pneumothorax |
| 195501 | 69878008 | Polycystic ovaries |
| 4153877 | 269406001 | Post-traumatic wound infection |
| 434319 | 44001008 | Premature ejaculation |
| 373478 | 41256004 | Presbyopia |
| 199876 | 73998008 | Prolapse of female genital organs |
| 4295888 | 76641005 | Prolapse of intestine |
| 194997 | 9713002 | Prostatitis |
| 4146239 | 267802000 | Pruritus of genital organs |
| 4285569 | 68633000 | Pupillary disorder |
| 81336 | 57773001 | Rectal prolapse |
| 380395 | 314407005 | Retinal dystrophy |
| 141825 | 267369002 | Simple goiter |
| 137054 | 201066002 | Skin striae |
| 434630 | 3745000 | Sleep-wake schedule disorder |
| 4195698 | 67801009 | Tenosynovitis |
| 4339088 | 87860000 | Testicular mass |
| 133141 | 6020002 | Tinea pedis |
| 440814 | 70070008 | Torticollis |
| 435140 | 67426006 | Toxic effect of alcohol |
| 4270490 | 62994001 | Tracheitis |
| 4028970 | 13617004 | Tracheobronchitis |
| 193326 | 87557004 | Urge incontinence of urine |
| 4092565 | 24976005 | Uterine prolapse |
| 140641 | 57019003 | Verruca vulgaris |
| 197036 | 197811007 | Vesicoureteric reflux |
| 261326 | 75570004 | Viral pneumonia |

| **Table S3. Baseline characteristics of NSAIDs in the analysis of aspirin, metformin and statin**   \| Characteristic,% \| After propensity score adjustment \| \| \| \| \| \| \| \| \| \| --- \| --- \| --- \| --- \| --- \| --- \| --- \| --- \| --- \| --- \| \| Aspirin  (n=31,839) \| Non-aspirin  (n=31,839) \| SMD \| Metformin  (n=6,764) \| Non-metformin  (n=6,764) \| SMD \| Statin  (n=10,251) \| Non-statin  (n=10,251) \| SMD \| \| **NSAID** \|  \|  \|  \|  \|  \|  \|  \|  \|  \| \| Ibuprofen \| 11.1 \| 11.1 \| 0.00 \| 10.4 \| 10.5 \| 0.00 \| 10.4 \| 10.6 \| -0.01 \| \| Naproxen \| 2.2 \| 2.2 \| 0.01 \| 2.4 \| 2.2 \| 0.01 \| 2.1 \| 2.2 \| -0.01 \| \| Fenoprofen \| 0.5 \| 0.5 \| 0.00 \| 0.4 \| 0.5 \| -0.01 \| 0.5 \| 0.4 \| 0.00 \| \| Ketoprofen \| 7.8 \| 7.6 \| 0.01 \| 7.6 \| 7.6 \| 0.00 \| 6.9 \| 6.9 \| 0.00 \| \| Flurbiprofen \| 0.4 \| 0.5 \| 0.00 \| 0.5 \| 0.4 \| 0.02 \| 0.5 \| 0.5 \| 0.00 \| \| Oxaprozin \| 0.3 \| 0.3 \| 0.00 \| 0.2 \| 0.4 \| -0.03 \| 0.2 \| 0.2 \| 0.00 \| \| Loxoprofen \| 26.8 \| 26.5 \| 0.01 \| 27.7 \| 27.0 \| 0.01 \| 28.0 \| 28.7 \| -0.02 \| \| Indomethacin \| 0.2 \| 0.3 \| 0.00 \| 0.2 \| 0.1 \| 0.02 \| 0.4 \| 0.3 \| 0.01 \| \| Sulindac \| 0.5 \| 0.4 \| 0.01 \| 0.5 \| 0.4 \| 0.01 \| 0.5 \| 0.3 \| 0.02 \| \| Etodolac \| 2.7 \| 2.6 \| 0.00 \| 2.6 \| 2.5 \| 0.01 \| 2.5 \| 2.6 \| 0.00 \| \| Ketorolac \| 2.7 \| 2.6 \| 0.00 \| 3.2 \| 3.4 \| -0.01 \| 3.4 \| 3.6 \| -0.01 \| \| Diclofena \| 29.1 \| 29.0 \| 0.00 \| 28.9 \| 28.4 \| 0.01 \| 28.2 \| 28.5 \| -0.01 \| \| Aceclofenac \| 22.4 \| 22.4 \| 0.00 \| 23.5 \| 23.0 \| 0.01 \| 23.8 \| 23.2 \| 0.01 \| \| Nabumetone \| 2.3 \| 2.4 \| -0.01 \| 2.2 \| 2.2 \| 0.00 \| 2.8 \| 2.8 \| 0.00 \| \| Piroxicam \| 11.3 \| 11.1 \| 0.01 \| 11.1 \| 11.1 \| 0.00 \| 11.0 \| 11.1 \| 0.00 \| \| Meloxicam \| 8.4 \| 8.0 \| 0.01 \| 8.4 \| 8.1 \| 0.01 \| 9.4 \| 9.3 \| 0.00 \| \| Lornoxicam \| 1.0 \| 1.0 \| 0.00 \| 0.7 \| 0.8 \| -0.01 \| 0.9 \| 0.7 \| 0.01 \| \| Tolfenamic acid \| 0.4 \| 0.4 \| 0.01 \| 0.3 \| 0.3 \| -0.01 \| 0.3 \| 0.4 \| -0.02 \| \| Celecoxib \| 1.2 \| 1.1 \| 0.00 \| 1.8 \| 1.4 \| 0.02 \| 1.9 \| 1.8 \| 0.01 \| \| Rofecoxib \| 0.2 \| 0.2 \| -0.01 \| 0.2 \| 0.3 \| -0.01 \| 0.2 \| 0.2 \| 0.00 \| \| Nimesulide \| 4.8 \| 4.8 \| 0.00 \| 4.5 \| 4.0 \| 0.03 \| 4.3 \| 4.3 \| 0.00 \|   **Table S4. Study characteristics included in the meta-analysis of aspirin and gastric cancer development** | | | | | | | | |
| --- | --- | --- | --- | --- | --- | --- | --- | --- | --- | --- | --- | --- | --- | --- | --- | --- | --- | --- | --- | --- | --- | --- | --- | --- | --- | --- | --- | --- | --- | --- | --- | --- | --- | --- | --- | --- | --- | --- | --- | --- | --- | --- | --- | --- | --- | --- | --- | --- | --- | --- | --- | --- | --- | --- | --- | --- | --- | --- | --- | --- | --- | --- | --- | --- | --- | --- | --- | --- | --- | --- | --- | --- | --- | --- | --- | --- | --- | --- | --- | --- | --- | --- | --- | --- | --- | --- | --- | --- | --- | --- | --- | --- | --- | --- | --- | --- | --- | --- | --- | --- | --- | --- | --- | --- | --- | --- | --- | --- | --- | --- | --- | --- | --- | --- | --- | --- | --- | --- | --- | --- | --- | --- | --- | --- | --- | --- | --- | --- | --- | --- | --- | --- | --- | --- | --- | --- | --- | --- | --- | --- | --- | --- | --- | --- | --- | --- | --- | --- | --- | --- | --- | --- | --- | --- | --- | --- | --- | --- | --- | --- | --- | --- | --- | --- | --- | --- | --- | --- | --- | --- | --- | --- | --- | --- | --- | --- | --- | --- | --- | --- | --- | --- | --- | --- | --- | --- | --- | --- | --- | --- | --- | --- | --- | --- | --- | --- | --- | --- | --- | --- | --- | --- | --- | --- | --- | --- | --- | --- | --- | --- | --- | --- | --- | --- | --- | --- | --- | --- | --- | --- | --- | --- | --- | --- | --- | --- | --- | --- | --- | --- | --- | --- | --- | --- | --- | --- | --- | --- | --- | --- | --- | --- | --- | --- | --- | --- | --- |
| Study label | Country | Study period | Study design | Source of database | Population | Number of patients | Definition of aspirin use | Newcastle-Ottawa scale (Selection / Comparability / Outcome) |
| 1999, Zaridze | Russia | 1993-1997 | Case-control | Moscow City Oncology Hospital and the Cancer Research Center | Not specified | 1058 | Use at least 2 days/week for ≥6 months | 3 / 2 / 2 |
| 2000, Coogan | USA | 1977-1998 | Case-control | Hospitals in Baltimore, Boston, New York, and Philadelphia | Not specified | 849 | Use at least 4 days/week for ≥3 months | 3 / 2 / 3 |
| 2001, Akre | Sweden | 1989-1995 | Case-control | Swedish Cancer Registry | Not specified | 1732 | Ever use of aspirin | 4 / 2 / 3 |
| 2005, Lindblad | UK | 1994-2001 | Case-control | General Practitioner Research Database | Not specified | 11023 | Ever use of aspirin | 4 / 2 / 3 |
| 2007, Fortuny | USA | 1980-2002 | Case-control | Group Health Cooperative and Henry Ford Health System's Health Alliance Plan | Not specified | 4492 | Ever use of aspirin | 3 / 2 / 3 |
| 2008, Duan | USA | 1992-1997 | Case-control | Los Angeles County Cancer Surveillance Program | Not specified | 2074 | Use of aspirin | 4 / 2 / 3 |
| 2009, Figueroa | USA | 1993-1995 | Case-control | Connecticut, New Jersey, and western Washington state residents | Not specified | 1062 | Use at least once a week for ≥6 months | 4 / 2 / 3 |
| 2010, Bertuccio | Italy | 1997-2007 | Case-control | Major Teaching and General Hospitals | Not specified | 772 | Use at least once a week for >6 months | 3 / 2 / 3 |
| 2012, Lee | Korea | 1999-2008 | Case-control | Samsung Medical Center Database | Patients with diabetes | 1966 | Use of aspirin (not further defined) | 3 / 2 / 3 |
| 2014, Gong | Korea | 2000-2010 | Case-control | Asan Medical Center Healthcare Center | Not specified | 654 | Use of aspirin (not further defined) | 3 / 2 / 2 |
| 2015, Wang | China | 2005-2010 | Case-control | The Second Hospital of Shenyang Medical College, the Hospital of Zhengzhou University, the First Hospital of China Medical University, and Liaoning Cancer Hospital | Not specified | 525 | Use at least once a week for ≥1 year | 3 / 2 / 3 |
| 2017, Iqbal | Taiwan | 2001-2011 | Case-control | Taiwan National Health Insurance Database | Not specified | 112870 | Use at least for 2 months during the 3-year period before the initial cancer diagnosis | 4 / 2 / 3 |
| 2019, Hsieh | Taiwan | 1997-2013 | Case-control | Taiwan National Health Insurance Research Database | Not specified | 197900 | Use > 28 cumulative defined daily dose | 4 / 2 / 3 |
| 2009, Abnet | USA | 1995-2003 | Cohort | NIH-AARP Diet and Health Study | Not specified | 311115 | Any use in the past 12 months | 3 / 2 / 3 |
| 2009, Epplein | USA | 1993-2004 | Cohort | The Multiethnic Cohort | Not specified | 169292 | Use at least 2 times/week for ≥1 months | 3 / 2 / 3 |
| 2013, Cook | USA | 1993-2012 | Cohort | Women's Health Study | Not specified | 39876 | 100 mg of aspirin every other day | 2 / 2 / 3 |
| 2018, Cheung | Hong Kong | 2003-2012 | Cohort | Clinical Data Analysis and Reporting System of the Hong Kong Hospital Authority | Patients who received *Helicobacter pylori* eradication therapy | 63605 | Ever use of aspirin | 3 / 2 / 3 |
| 2018, Kim | Korea | 2002-2013 | Cohort | National Health Insurance Services | Not specified | 461489 | Ever use of aspirin | 4 / 2 / 3 |

| **Table S5. Study characteristics included in the meta-analysis of metformin and gastric cancer development** | | | | | | | | |
| --- | --- | --- | --- | --- | --- | --- | --- | --- |
| Study label | Country | Period | Design | Source of database | Population | Number of patients | Definition of metformin use | Newcastle-Ottawa scale (Selection / Comparability / Outcome) |
| 2012, Ruiter | The Netherlands | 1998-2008 | Cohort | PHARMO Record Linkage System | Patients who took hypoglycemic agents | 85289 | Not specified | 4 / 2 / 3 |
| 2014, Kim | Korea | 2004-2010 | Cohort | Korea National Health Insurance claim database | Patients with type 2 diabetes | 32978 | Prescription of metformin ≥6 consecutive months | 4 / 2 / 3 |
| 2014, Tsilidis | UK | 1987-2011 | Cohort | Clinical Practice Research Datalink | Patients with type 2 diabetes | 69748 | Not specified | 4 / 2 / 3 |
| 2015, Valent | Italy | 2002-2014 | Cohort | Regional health information in the Friuli Venezia Giulia | Patients with type 2 diabetes | 109907 | Not specified | 4 / 2 / 3 |
| 2016, Tseng | Taiwan | 1999-2011 | Cohort | National Health Insurance Research Database in Taiwan | Patients with type 2 diabetes | 226056 | Ever use of metformin | 4 / 2 / 3 |
| 2017, de Jong | The Netherlands | 1998-2011 | Cohort | PHARMO Record Linkage System | Patients who took hypoglycemic agents | 35654 | Current use of metformin | 4 / 2 / 3 |
| 2018, Murff | USA | 2001-2008 | Cohort | National Veterans Health Administration databases | Patients who took hypoglycemic agents | 84434 | Continuous use with no gaps in metformin use >90 days | 3 / 2 / 3 |
| 2019, Cheung | Hong Kong | 2003-2015 | Cohort | Clinical Data Analysis and Reporting System of Hong Kong | Patients with diabetes who received *Helicobacter pylori* eradication therapy | 7266 | Use of metformin >180 days | 4 / 2 / 3 |
| 2019, Zheng | Sweden | 2005-2015 | Cohort | Swedish Prescribed Drugs and Health Cohort | Patients with diabetes | 544130 | Not specified | 4 / 2 / 3 |

| **Table S6. Study characteristics included in the meta-analysis of statin and gastric cancer development** | | | | | | | | | |
| --- | --- | --- | --- | --- | --- | --- | --- | --- | --- |
| Study label | Country | Period | Study design | Source of database | Population | Number of patients | Definition of statin use | Type of statin | Newcastle-Ottawa scale (Selection / Comparability / Outcome) |
| 2004, Graaf | Netherlands | 1985-1998 | Case-control | PHARMO Record Linkage System | Not specified | 20105 | Use of statin at least 6 months | Atorvastatin, pravastatin, simvastatin, fluvastatin, and cervastatin | 4 / 2 / 3 |
| 2004, Kaye | UK | 1990-2002 | Case-control | General Practice Research Database | Not specified | 18088 | Current user (at least 1 prescription during the yeaer before the index date) | Any statin | 4 / 2 / 3 |
| 2011, Vinogradova | UK | 1998-2008 | Case-control | QResearch database | Not specified | 10271 | Ever use | Atorvastatin, pravastatin, simvastatin, fluvastatin, rosuvastatin, and cerivastatin | 4 / 2 / 3 |
| 2012, Lee | Korea | 1999-2008 | Case-control | Samsung Medical Center | Patients with diabetes | 1966 | Use of statin at least 6 months | Atorvastatin, lovastatin, pravastatin, simvastatin, rosuvastatin, pitavastatin, and cerivastatin | 3 / 2 / 3 |
| 2016, Lin | Taiwan | 2005-2010 | Case-control | National Health Insurance Research Database in Taiwan | Not specified | 39455 | Ever use | Lovastatin and simvastatin | 4 / 2 / 3 |
| 2020, Busby | UK | 1999-2011 | Case-control | Primary Care Clinical Information Unit Research database | Not specified | 11653 | Ever use | Any statin | 4 / 2 / 3 |
| 2008, Friedman | USA | 1988-2003 | Cohort | Kaiser Permanente Medical Care Program in northern California | Not specified | 4243067 | Ever use | Any statin | 4 / 2 / 3 |
| 2010, Hippisley-Cox | England and Wales | 2002-2008 | Cohort | QResearch database | Not specified | 2121786 | Not specified | Atorvastatin, pravastatin, simvastatin, fluvastatin, and rosuvastatin | 4 / 2 / 3 |
| 2010, Matsushita | Japan | N/A | Cohort | MEGA study, KLIS, and Holicos-PAT | Patients with hypercholesterolemia | 13724 | Use of statin for at least 4 years | Pravastatin | 3 / 2 / 3 |
| 2020, Cheung | China | 2003-2015 | Cohort | Clinical Data Analysis and Reporting System | Patients who received *Helicobacter pylori* eradication therapy | 63605 | Use of statin at least 180 days | Atorvastatin, simvastatin, and rosuvastatin | 4 / 2 / 3 |
| 2020, You | Korea | 2002-2015 | Cohort | National Health Insurance Service-National Health Screening Cohort | Patients with hypercholesterolemia | 61149 | Ever use in the two years of enrollment period | Atorvastatin, lovastatin, pravastatin, simvastatin, fluvastatin, and cerivastatin | 4 / 2 / 3 |
| MEGA, Management of Elevated Cholesterol in the Primary Prevention Group of Adult Japanese; KLIS, Kyushu Lipid Intervention Study; Holicos-PAT, Hokuriku Lipid Coronary Heart Disease Study-Pravastatin Atherosclerosis Trial | | | | | | | | | |
