# Supplementary material for: Aspirin, metformin, and statin use on the risk of gastric cancer: A nationwide population‐based cohort study in Korea with systematic review and meta‐analysis
Source: Cancer Med. 2021 Dec 30;11(4):1217–31. doi: 10.1002/cam4.4514 (PMC8855895; doi:10.1002/cam4.4514)
Supplement: Supplementary file 10 — Appendix S1 [file CAM4-11-1217-s002.docx]

**Appendix 1. Detailed search strategy**

**1. Aspirin and gastric cancer**

The latest search date: 17 March 2020

Medline (PubMed)

(aspirin[tw] OR ASA[tw] OR (acetylsalicylic acid[tw]) OR acetylsalicylate[tw]) AND ((gastric cancer[tw]) OR (stomach cancer[tw]) OR (gastric carcinoma[tw]) OR (stomach carcinoma[tw]) OR (gastric adenocarcinoma[tw]) OR (stomach adenocarcinoma[tw])) AND (cohort[tw] OR cohorts[tw] OR (case-control[tw]) OR registry[tw] OR database[tw]) AND ("1980/01/01"[Date - Publication] : "3000"[Date - Publication]) NOT review[Publication Type] NOT meta-analysis[Publication Type]

Embase (Ovid)

#1: ((aspirin or ASA or 'acetylsalicylic acid' or acetylsalicylate) and ('gastric cancer' or 'stomach cancer' or 'gastric carcinoma' or 'stomach carcinoma' or 'gastric adenocarcinoma' or 'stomach adenocarcinoma') and (cohort or cohorts or 'case-control' or registry or database)).ab,ti.

#2: Limit 1 to (english language and embase and yr="1980 -Current" and (article or article in press))

Cochrane library

#1: aspirin or ASA or 'acetylsalicylic acid' or acetylsalicylate

#2: 'gastric cancer' or 'stomach cancer' or 'gastric carcinoma' or 'stomach carcinoma' or 'gastric adenocarcinoma' or 'stomach adenocarcinoma'

#3: cohort or cohorts or 'case-control' or registry or database

#4: #1 and #2 and #3 (with Publication Year from 1980 to 2020, in Trials)

**2. Metformin and gastric cancer**

The latest search date: 05 April 2020

Medline (PubMed)

(metformin[tw] OR biguanide[tw] OR dimethylbiguanide[tw] OR dimethylguanylguanidine[tw] OR glucophage[tw]) AND ((gastric cancer[tw]) OR (stomach cancer[tw]) OR (gastric carcinoma[tw]) OR (stomach carcinoma[tw]) OR (gastric adenocarcinoma[tw]) OR (stomach adenocarcinoma[tw])) AND (cohort[tw] OR cohorts[tw] OR (case-control[tw]) OR registry[tw] OR database[tw]) AND ("1980/01/01"[Date - Publication] : "3000"[Date - Publication]) NOT review[Publication Type] NOT meta-analysis[Publication Type]

Embase (Ovid)

#1: ((metformin or biguanidie or dimethylbiguanide or dimethylguanylguanidine) and ('gastric cancer' or 'stomach cancer' or 'gastric carcinoma' or 'stomach carcinoma' or 'gastric adenocarcinoma' or 'stomach adenocarcinoma') and (cohort or cohorts or 'case-control' or registry or database)).ab,ti.

#2: Limit 1 to (english language and embase and yr="1980 -Current" and (article or article in press))

Cochrane library

#1: metformin or biguanidie or dimethylbiguanide or dimethylguanylguanidine

#2: 'gastric cancer' or 'stomach cancer' or 'gastric carcinoma' or 'stomach carcinoma' or 'gastric adenocarcinoma' or 'stomach adenocarcinoma'

#3: cohort or cohorts or 'case-control' or registry or database

#4: #1 and #2 and #3 (with Publication Year from 1980 to 2020, in Trials)

**3. Statin and gastric cancer**

The latest search date: 20 April 2020

Medline (PubMed)

(statin[tw] OR (3-Hydroxy-3-Methylglutaryl Coenzyme A[tw]) OR (HMG-CoA[tw])) AND ((gastric cancer[tw]) OR (stomach cancer[tw]) OR (gastric carcinoma[tw]) OR (stomach carcinoma[tw]) OR (gastric adenocarcinoma[tw]) OR (stomach adenocarcinoma[tw])) AND (cohort[tw] OR cohorts[tw] OR (case-control[tw]) OR registry[tw] OR database[tw]) AND ("1980/01/01"[Date - Publication] : "3000"[Date - Publication]) NOT review[Publication Type] NOT meta-analysis[Publication Type]

Embase (Ovid)

#1: ((statin or '3-Hydroxy-3-Methylglutaryl Coenzyme A' or 'HMG-CoA') and ('gastric cancer' or 'stomach cancer' or 'gastric carcinoma' or 'stomach carcinoma' or 'gastric adenocarcinoma' or 'stomach adenocarcinoma') and (cohort or cohorts or 'case-control' or registry or database)).ab,ti.

#2: Limit 1 to (english language and embase and yr="1980 -Current" and (article or article in press))

Cochrane library

#1: statin or "3-Hydroxy-3-Methylglutaryl Coenzyme A" or "HMG-CoA"

#2: 'gastric cancer' or 'stomach cancer' or 'gastric carcinoma' or 'stomach carcinoma' or 'gastric adenocarcinoma' or 'stomach adenocarcinoma'

#3: cohort or cohorts or 'case-control' or registry or database

#4: #1 and #2 and #3 (with Publication Year from 1980 to 2020, in Trials)
